# Epidemiology of Coronavirus Disease 2019 in US Immigration and Customs Enforcement Detention Facilities

**DOI:** 10.1001/jamanetworkopen.2020.34409

**Published:** 2021-01-19

**Authors:** Felicia O. Casanova, Alice Hamblett, Lauren Brinkley-Rubinstein, Kathryn M. Nowotny

**Affiliations:** 1 Institute for Advanced Study of the Americas, Department of Sociology, University of Miami, Coral Gables, Florida; 2 Brown University School of Public Health, Providence, Rhode Island; 3 Center for Health Equity Research, Department of Social Medicine, University of North Carolina at Chapel Hill; 4 Department of Sociology, University of Miami, Coral Gables, Florida

## Abstract

This cohort study describes the burden of coronavirus disease 2019 among people detained by US Immigration and Customs Enforcement compared with the US population.

## Introduction

The US is facing a humanitarian crisis as tens of thousands of people are held in detention centers under Immigration and Customs Enforcement (ICE). Practices undertaken by ICE, such as detainment, deportation, and searches, adversely affect the physical and mental health of those who are undocumented.^1^ Immigration and Customs Enforcement facilities have been characterized as unsanitary, unsafe, and inhumane by a recent whistleblower.^2^ Home to moldy, uncleaned bathrooms and limited personal hygiene supplies and medical services, facilities pose health risks to people even beyond the context of a global pandemic.^3^ Human rights advocates have called for the release of people detained and the suspension of deportation flights.^4^ Thus far, ongoing deportation flights have led to documented spread of coronavirus disease 2019 (COVID-19) in more than 11 countries.^5^ Herein, we describe the COVID-19 burden among people detained by ICE compared with the US population.

## Methods

Data concerning ICE and COVID-19 for this cohort study were obtained from the COVID Prison Project from May 5 to September 15, 2020, with the ICE mean daily population (MDP) serving as the denominator. General population COVID-19 data were obtained from *The New York Times*, with denominator data from the American Community Survey (eMethods in the Supplement). We used publicly available data and were exempted from institutional review board approval and informed consent by University of North Carolina at Chapel Hill. We followed the Strengthening the Reporting of Observational Studies in Epidemiology (STROBE) reporting guidelines.

We used baseline facility MDP to calculate cumulative case rates per 1000 persons detained by ICE over time and compared this with cumulative case rates in the general population. We also used ICE testing and case data to calculate the percentage tested and test positivity rate (percentage of tests returned with positive results). For county-level comparisons, we used year-to-date MDP from September 12, 2020, to calculate cumulative and current case rates for ICE designated facilities. County cumulative case rates in the general population were also calculated. Risk ratios compared cumulative case rates in facilities and the corresponding county. Analysis was performed using STATA, version 15 (StataCorp LLC) and Excel (Microsoft Corporation).

## Results

One hundred sixty-seven facilities housed people detained by ICE, most of which were facilities that primarily housed non-ICE detainees (eg, county jails). Immigration and Customs Enforcement reported at least 1 COVID-19 case in 96 facilities. Using baseline MDP, the overall September 15 cumulative case rate was 214 per 1000 people (5810 cases among 27 189 people); ICE reported 6 deaths. There were 28 designated ICE facilities that exclusively housed people detained by ICE, including 4 family residential centers.

As testing rates reported by ICE increased, case rates increased, and test positivity rates decreased (Figure). However, the increase in case rates among people detained by ICE has outpaced the growth in the US population. The cumulative case rate in the 28 ICE-designated facilities varied from 0 per 1000 to 1050 per 1000 at Webb County Detention Center in Texas (Table). The risk ratio was greater than 1 in 20 of 28 facilities.

**Figure.**
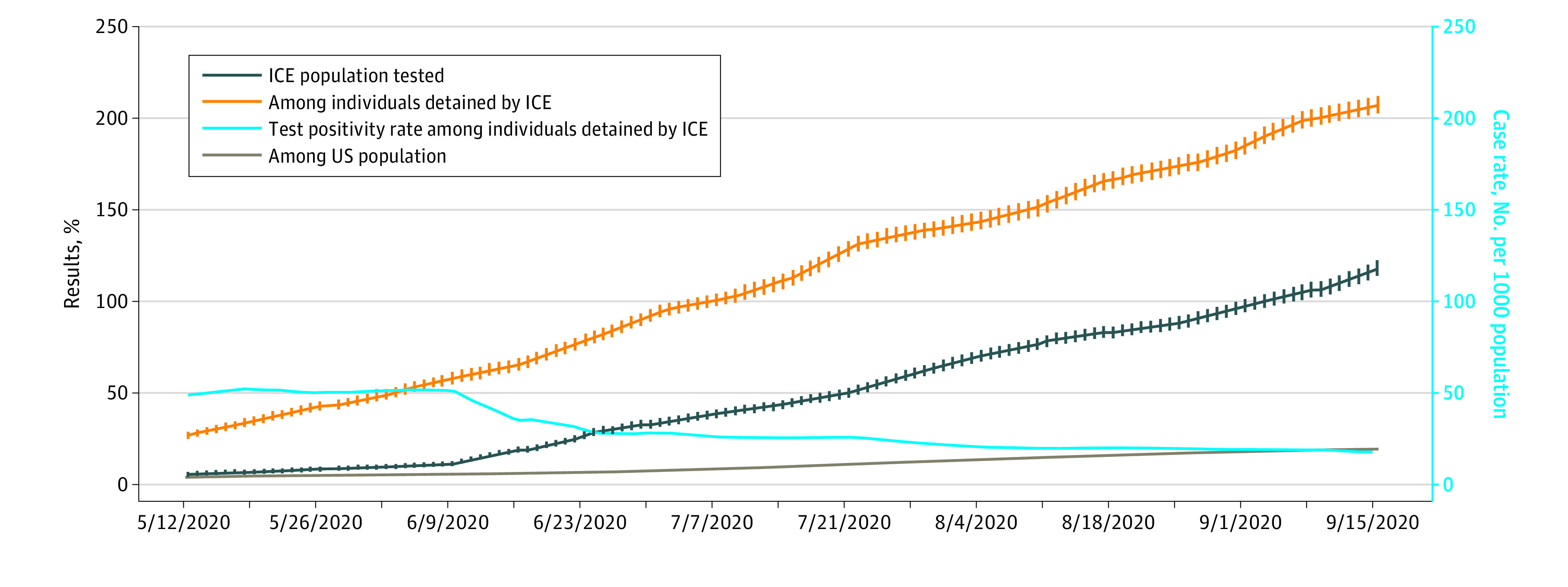
Testing for and Cases of Coronavirus Disease 2019 in Immigration and Customs Enforcement (ICE) Facilities

**Table.** COVID-19 Case Rates in Designated ICE Facilities Compared With County Case Rates Per 1000 as of September 15, 2020

Facility name, state abbreviation (population)	County name (population)	ICE designation^a^	ICE cumulative case rate		County cumulative case rate		RR for cumulative case rates (95% CI)	ICE current case rate^b^	
			No. of cases	Rate (95% CI)	No. of cases	Rate (95% CI)		No. of cases	Rate (95% CI)
Adams County Detention Center, Mississippi (908)	Adams (30 693)	DIGSA	78	86 (68-104)	877	29 (27-30)	3.01 (2.41-3.75)	14	15 (7-23)
Adelanto ICE Processing Center, California (1330)	San Bernardino (2 180 085)	DIGSA	14	11 (5-16)	50 978	23 (23-24)	0.45 (0.27-0.76)	1	1 (0-2)
Berks County Family Shelter, Pennsylvania (37)	Berks (421 164)	FRC	0	0	6641	16 (15-16)	0	0	0
Buffalo (Batavia) Service Processing Center, New York (374)	Genesee (57 280)	SPC	49	131 (97-165)	307	5 (5-6)	24.44 (18.40-32.47)	0	0
Caroline Detention Facility, Virginia (230)	Caroline (30 725)	DIGSA	7	30 (8-53)	299	10 (9-11)	3.13 (1.49-6.54)	2	9 (0-21)
El Paso Service Processing Center, Texas (534)	El Paso (839 238)	SPC	190	356 (315-396)	21 826	26 (26-26)	13.68 (12.20-15.35)	5	9 (1-18)
Elroy Federal Contract Facility, Arizona (1036)	Pinal (462 789)	DIGSA	250	241 (215-267)	10 198	22 (22-22)	10.95 (9.81-12.22)	1	1 (0-3)
Florence Service Processing Center, Arizona (274)^c^	Pinal (462 789)	SPC	0	0	10 198	22 (22-22)	0	0	0
Folkston IPC (Main), Georgia (202)^d^	Charlton (13 392)	DIGSA	67	332 (267-397)	599	45 (41-48)	7.42 (6.01-9.16)	18	89 (50-128)
Immigration Centers of American Farmville, Virginia (507)	Prince Edward (22 802)	DIGSA	339	669 (628-710)	541	24 (22-26)	28.18 (25.41-31.25)	0	0
Jackson Parish Correctional Center, Louisiana (623)	Jackson (15 744)	DIGSA	73	117 (92-142)	586	37 (34-40)	3.15 (2.50-3.96)	67	108 (83-132)
Karnes County Residential Center, Texas (218)	Karnes (15 601)	FRC	79	362 (299-426)	753	48 (45-52)	7.51 (6.21-9.07)	0	0
Krome North Service Processing Center, Florida (542)	Miami-Dade (2 716 940)	SPC	182	336 (296-376)	164 687	61 (60-61)	5.54 (4.92-6.24)	2	4 (0-9)
La Palma Correctional Center, Arizona (1004)	Pinal (462 789)	DIGSA	366	365 (335-394)	10 198	22 (22-22)	16.54 (15.21-17.99)	11	11 (5-17)
Laredo Processing Center, Texas (202)	Webb (276 652)	DIGSA	6	30 (6-53)	12 733	46 (45-47)	0.65 (0.29-1.42)	5	25 (3-46)
LaSalle ICE Processing Center–Jenna, Louisiana (977)	LaSalle (7520)	DIGSA	29	30 (19-40)	415	55 (50-60)	0.54 (0.37-0.78)	2	2 (0-5)
Otero County Processing Center, New Mexico (646)	Dona Ana (218 195)	DIGSA	152	235 (203-268)	3049	14 (13-14)	16.84 (14.59-19.44)	2	3 (0-7)
Pine Prairie ICE Detention Facility, Louisiana (523)	Evangeline (33 395)	DIGSA	65	124 (96-153)	1270	38 (36-40)	3.27 (2.59-4.13)	0	0
Port Isabel, Texas (708)	Cameron (423 163)	SPC	145	205 (175-235)	22 222	53 (52-53)	3.90 (3.37-4.51)	3	4 (0-9)
Prairieland Detention Facility, Texas (493)	Johnson (175 817)	DIGSA	88	178 (145-212)	2699	15 (15-16)	11.63 (9.59-14.10)	16	32 (17-48)
Richwood Correctional Center, Louisiana (507)	Ouachita (153 279)	DIGSA	110	217 (181-253)	5896	38 (38-39)	5.64 (4.77-6.67)	37	73 (50-96)
River Correctional Center, Louisiana (294)	Concordia (19 259)	DIGSA	22	75 (45-105)	497	26 (24-28)	2.90 (1.92-4.37)	9	31 (11-50)
South Louisiana Detention Center, Louisiana (433)	Iberville (32 511)	DIGSA	3	7 (0-15)	1391	43 (41-45)	0.16 (0.05-0.50)	0	0
South Texas Family Residential Center, Texas (770)	Frio (20 306)	FRC	4	5 (0-10)	666	33 (30-35)	0.16 (0.06-0.42)	0	0
Stewart Detention Center, Georgia (1381)	Stewart (6621)	DIGSA	338	245 (222-267)	414	63 (57-68)	3.91 (3.43-4.46)	26	19 (12-26)
T. Don Hutto Residential Center, Texas (293)	Williamson (590 551)	FRC	0	0	8446	14 (14-15)	0.00 (0.00-0.00)	0	0
Webb County Detention Center, Texas (80)	Webb (276 652)	DIGSA	84	1050 (1028-1072)	12 733	46 (45-47)	22.81 (22.43-23.20)	0	0
Winn Correctional Center, Louisiana (1040)	Winn (13 904)	DIGSA	202	194 (170-218)	598	43 (40-46)	4.52 (3.90-5.23)	36	35 (24-46)

Abbreviations: DIGSA, Dedicated Intergovernmental Service Agreement; FRC, Family Residential Center; ICE, Immigration and Customs Enforcement; RR, risk ratio; SPC, Service Processing Center.

^a^ DIGSA is a publicly owned facility operated by state/local government(s) or private contractors in which ICE contracts to use all bed space or facilities that house only ICE detainees. FRC is a facility that accommodates and cares for family units who remain together while awaiting their proceedings, and SPC is a facility owned by the government.

^b^ Current Cases: COVID-19 positive cases currently in custody under isolation or monitoring.

^c^ This facility is separate from Florence Correctional Center. The Florence Correctional Complex serves a number of government agencies. There are reported COVID-19 cases among ICE detainees at this facility.

^d^ There is a separate annex facility at this location, which is excluded from the population denominator.

## Discussion

Cumulative case rates among people detained by ICE are higher than those of the US population and dwarf those of surrounding communities. However, this study has limitations. This analysis depends on ICE reporting; thus, cases may actually be higher.^6^ With a mean stay of 38 days, it is difficult to assess mortality and testing rates given high population churn. We report crude rates because age data for ICE detainees are not available. It is likely that the age structure is younger than that of the general population. Facility staff were excluded. There are potential differences in facility responses to COVID-19 (https://www.freedomforimmigrants.org/map). Ultimately, it is imperative that expeditious action is taken to protect people housed in ICE detention facilities from COVID-19 by reducing the number of people detained and terminating raids, transfers, and deportation flights.

## Notes

Supplement. **eMethods.** Data Description and Sources
