# Supplementary material for: Epidemiology of Coronavirus Disease 2019 in US Immigration and Customs Enforcement Detention Facilities
Source: JAMA Netw Open. 2021 Jan 19;4(1):e2034409. doi: 10.1001/jamanetworkopen.2020.34409 (PMC7816105; doi:10.1001/jamanetworkopen.2020.34409)
Supplement: Supplement. — eMethods. Data Description and Sources [file jamanetwopen-e2034409-s001.pdf]

Supplementary Online Content

Casanova FO, Hamblett A, Brinkley-Rubinstein L, Nowotny KM. Epidemiology of coronavirus disease 2019 in US immigration and customs enforcement detention facilities. *JAMA Netw Open*. 2021;4(1):e2034409. doi:10.1001/jamanetworkopen.2020.34409

eMethods. Data Description and Sources

This supplementary material has been provided by the authors to give readers additional information about their work.

©2021 Casanova FO et al. *JAMA Network Open*.

eMethods. Data Description and Sources

The COVID-19 data on cases and testing for Immigration and Customs Enforcement (ICE) detention facilities were from COVID Prison Project (CPP) as of September 15, 2020: https://covidprisonproject.com. CPP publishes a daily aggregate dataset examining COVID-19 in correctional facilities, including data on the number of tests, the number of confirmed positive cases, and mortality, among other factors, due to COVID-19 among correctional staff and incarcerated individuals. The data were aggregated by CPP based on public reports by prison systems. Each day, counts were extracted from Departments of Correction websites and supplemented with information from media reports and press releases. In total, CPP collects daily data for 53 U.S. prison systems (all 50 states, Puerto Rico, the Federal Bureau of Prisons, and ICE). Only data for ICE were used in this paper. ICE does report COVID-19 case data for staff; however, staff population denominator data were not available. Therefore, staff were excluded from this paper.

The ICE population data year-to-date ADP as of September 12, 2020 were from ICE detention statistics: https://www.ice.gov/detention-management. This link provided a direct download: https://www.ice.gov/doclib/detention/FY20-detentionstats.xlsx. The case rates per 1,000 in Figure 1 used baseline population data--year-to-date ADP for May 2020. The case rates per 1,000 in Table 1 used the most recent year-to-date ADP reported at the facility level.

The general population COVID-19 data were from The New York Times as of September 15, 2020: https://www.nytimes.com/interactive/2020/us/coronavirus-us-cases.html. This link provided direct downloads for data files: https://github.com/nytimes/covid-19-data/find/master. The U.S. file (https://github.com/nytimes/covid-19-data/blob/master/us.csv) was used for Figure 1. The county file was used for Table 1 (https://github.com/nytimes/covid-19-data/blob/master/us-counties.csv). County-level population data were from 2019 American Community Survey estimates: https://www.census.gov/data/datasets/time-series/demo/popest/2010s-counties-total.html.

©2021 Casanova FO et al. *JAMA Network Open*.

ICE detention facilities and counties were matched using Federal Information Processing Standard (FIPS) codes and county names. ICE detention facility zip codes were matched to county names and FIPS codes. These were then matched to data from the New York Times and the American Community Survey.

©2021 Casanova FO et al. *JAMA Network Open*.
